# An Assessment of Mushroom Consumption on Cardiometabolic Disease Risk Factors and Morbidities in Humans: A Systematic Review

**DOI:** 10.3390/nu15051079

**Published:** 2023-02-21

**Authors:** Cassi N. Uffelman, Nok In Chan, Eric M. Davis, Yu Wang, Bethany S. McGowan, Wayne W. Campbell

**Affiliations:** 1 Department of Nutrition Science, Purdue University, West Lafayette, IN 47907, USA; 2 Library of Engineering and Science, Purdue University, West Lafayette, IN 47907, USA

**Keywords:** hypertension, dyslipidemia, hyperglycemia, inflammation, cardiovascular disease, diabetes mellitus, *Agaricus bisporus*, *Pleurotus ostreatus*

## Abstract

Mushrooms, unique edible fungi, contain several essential nutrients and bioactive compounds which may positively influence cardiometabolic health. Despite a long history of consumption, the health benefits of mushrooms are not well documented. We conducted a systematic review to assess the effects of and associations between mushroom consumption and cardiometabolic disease (CMD)-related risk factors and morbidities/mortality. We identified 22 articles (11 experimental and 11 observational) from five databases meeting our inclusion criteria. Limited evidence from experimental research suggests mushroom consumption improves serum/plasma triglycerides and hs-CRP, but not other lipids, lipoproteins, measures of glucose control (fasting glucose and HbA1c), or blood pressure. Limited evidence from observational research (seven of 11 articles with *a posteriori* assessments) suggests no association between mushroom consumption and fasting blood total or LDL cholesterol, glucose, or morbidity/mortality from cardiovascular disease, coronary heart disease, or type 2 diabetes mellitus. Other CMD health outcomes were deemed either inconsistent (blood pressure, HDL cholesterol, and triglycerides) or insufficient (HbA1c/hyperglycemia, hs-CRP, cerebrovascular disease, and stroke). The majority of the articles vetted were rated “poor” using the NHLBI study quality assessment tool due to study methodology and/or poor reporting issues. While new, high-quality experimental and observational research is warranted, limited experimental findings suggest greater mushroom consumption lowers blood triglycerides and hs-CRP, indices of cardiometabolic health.

## 1. Introduction

Mushrooms are a unique food source, distinct from plant and animal foods, that are generally considered healthful. Mushrooms are low in energy, fat-free, cholesterol-free, and very low in sodium, which supports many of the current recommendations set forth by the Dietary Guidelines for Americans [1,2]. Along with several essential nutrients, including selenium, potassium, and B vitamins, mushrooms also contain bioactive compounds which may elicit health benefits. While the concentrations of bioactive compounds vary among mushroom species, the most commonly consumed species, *Agaricus bisporus*, includes appreciable amounts of beta-glucans, lovastatin, L-ergothioneine, ergosterol, and polyphenols [1,3,4,5]. Beta-glucans and lovastatin are known for having cholesterol-lowering properties that may reduce one’s risk of developing cardiovascular disease. L-ergothioneine is a diet-derived amino acid with antioxidant and anti-inflammatory properties associated with the development of multiple degenerative and chronic diseases, including several cardiometabolic diseases (CMD) [6,7]. Importantly, L-ergothioneine is not synthesized by animals or higher plants but is biosynthesized by mushrooms, cyanobacteria, and some soil bacteria. Low levels of L-ergothioneine are found in several foods, but the greatest dietary sources are mushrooms [5,8,9,10]. Mushrooms also contain ergosterol, the precursor for vitamin D2, which, when exposed to ultraviolet rays, promotes the conversion of ergosterol to vitamin D2, making them a natural source of vitamin D for humans [11]. Additionally, ultraviolet-irradiation of mushrooms may generate stress in fungal cell’s metabolic pathways, which leads to the increased synthesis of several other bioactive compounds (secondary metabolites), including beta-glucans, phenolics, and L-ergothioneine, among others [12]. These findings suggest additional advantages of ultraviolet irradiation to enhance the nutritional quality of mushrooms. Taken together, the distinct chemical composition of mushrooms suggests their potential as a functional food for health [13].

Narrative reviews [12,14,15] describe multiple mushroom-derived bioactive compounds associated with cardiometabolic health. Identified primarily using cell and animal models, the cardioprotective effects of mushrooms include hypolipidemic/hypocholesterolemic, hypotensive, and anti-atherogenic [14]. While the mechanisms of action have not been fully elucidated, hypocholesterolemic properties are associated with lovastatin, which inhibits HMG-CoA reductase, the enzyme required to produce cholesterol [14]. Hypotensive effects of various mushroom species, including *Ganoderma lingzhi* and *Pleurotus pulmonarius,* are attributed to endogenous proteases that act as Angiotensin-Converting Enzyme (ACE) inhibitors *in vivo* [16,17]. L-ergothioneine has exhibited cardioprotective effects in an *in vitro* model of atherogenesis demonstrated by reduced expression of adhesion molecules (intercellular adhesion molecule-1 [ICAM-1]; vascular cell adhesion molecule-1 [VCAM-1]; endothelial-leukocyte adhesion molecule-1 [E-selectin]) and reduced monocyte binding to human aortic endothelial cells [18]. The anti-diabetic properties of isolated compounds derived from mushrooms, including polysaccharides such as beta-glucans, have hypoglycemic properties in diabetic mice/rats [15]. Of note, the majority of articles included in these narrative reviews studied compounds isolated from mushrooms and which may have been at pharmacological doses (not in concentrations found in whole, dietary mushrooms). Nonetheless, these findings suggest plausible mechanisms of action for the role of mushrooms in promoting cardiometabolic health.

Previous systematic reviews suggest mushrooms, including *Agaricus bisporus* and *Pleurotus ostreatus*, may positively influence several risk factors for cardiometabolic diseases. Consumption of whole or processed *Agaricus bisporus* mushrooms has favorable health effects on glucose, lipids (total cholesterol, HDL- and LDL-cholesterol, and triglycerides), and several markers of inflammation, including TNF-α, adiponectin, and oxygen radical absorbance capacity (ORAC) [19]. Another recent systematic review described the consumption of *Pleurotus ostreatus* improved fasting and/or postprandial glucose, total cholesterol, LDL-cholesterol, and/or triglycerides in eight clinical trials [20]. Consistent with these reviews, favorable effects of mushroom consumption (species not specified) on total, LDL- and HDL-cholesterol, and triglycerides have been described. The authors also stated mushroom consumption is probably associated with reduced blood pressure [21].

While these previous systematic reviews [19,20,21] report generally positive impacts of mushroom consumption on indices of cardiometabolic health, the current systematic review, to our knowledge, is the first to more comprehensively assess the cardiometabolic impacts of mushroom consumption (of all species) using evidence from both experimental and observational research. Therefore, the purpose of this systematic review is to assess the effects of and associations between whole mushroom consumption—inclusive of fresh or dried—and cardiometabolic disease risk factors and morbidities/mortality in adults using data from peer-reviewed randomized controlled trials (RCTs) and observational studies. This systematic review does not assess the effects of mushroom-derived or isolated compounds on the outcomes of interest.

## 2. Methods

### 2.1. Experimental Design

This systematic review was registered at the International Prospective Registrar of Systematic Reviews (PROSPERO) before database searches commenced (PROSPERO Registration ID # CRD42021214441). This systematic review meets the Preferred Reporting Items for Systematic review and Meta-Analysis Protocols (PRISMA-P) 2015 checklist guidelines [22].

### 2.2. Inclusion and Exclusion Criteria

The Population, Intervention, Comparison, Outcome, and Study Type (PICOS) criteria defining our research question are presented in Table 1. Inclusion criteria were: English language; human adults age ≥ 18 y; whole mushroom consumption (fresh or dried) of any species; reporting on at least 1 primary outcome; peer-reviewed randomized controlled trial or observational study. Exclusion criteria were: not available in English; participant age < 18 y; no reported mushroom consumption or statistically significant differences in mushroom consumption; mushrooms not in whole form (i.e., an extract from mushrooms, consumed in the form of a capsule); no outcomes of interest reported; animal or cell model studies; full article not available; not original research (review articles, commentaries, grey literature).

### 2.3. Search Strategy and Article Screening

A search of five databases, including PubMed, Cumulative Index to Nursing and Allied Health Literature (CINAHL), Web of Science, Scopus, and Cochrane library, was conducted on 28 July 2021 and updated on 15 July 2022. Search strategies were created by a research librarian from Purdue University’s Library of Engineering and Science for each database (Table 2). Two researchers independently screened and cross-checked articles at all stages to determine their eligibility. In the first pass, researchers independently identified potentially relevant articles by their titles and abstracts. Article eligibility was confirmed in the second pass full-text screening. Any disagreements during the first or second pass screenings were discussed between each pair of researchers and sent to a third reviewer if no consensus was made.

### 2.4. Quality Assessment

We used the National Heart, Lung, and Blood Institute’s (NHLBI) study quality assessment tool to assess the internal validity of each included article [23]. Briefly, four different versions of this tool were used to assess the study quality for controlled intervention studies, pre-post studies without a control group, observational cohort and cross-sectional studies, and case-control studies. Each version of the NHLBI study quality assessment tool consists of 12–14 questions which are designed to help reviewers assess key components of the study related to study methodology and implementation. The NHLBI tool does not generate a quantitative score rating. Instead, a rating of “good”, “fair”, or “poor” was assigned based on a critical appraisal of study characteristics that are most pertinent to high-quality research studies (i.e., allocation bias, differences in baseline characteristics, high differential dropout rates, completers analysis rather than intention-to-treat analysis, selection bias, measurement bias, etc.). Each article was independently assessed by two researchers and cross-checked for accuracy. Any discrepancies in the cross-check were discussed until a consensus was reached. The two researchers then agreed on a final rating for each included article.

### 2.5. Effect Measures, Calculations, and Synthesis Methods

We found insufficient data (raw mean change and/or variance of change) in the literature to complete statistical analyses. Rather than conducting a meta-analysis, we are limited to a qualitative report of the results. The results in this systematic review are presented based on statistical significance (increase, decrease, no change) reported in the original manuscript, not on clinical significance. Supplementary Material tables include results from the original research articles presented as mean change and variance (standard deviation or 95% confidence interval, when applicable). The mean change was estimated if baseline and post values only were reported. All change values and variances were rounded to the nearest tenth. Conversions of numeric values from the original manuscript were as follows: (1) standard error was converted to standard deviation ($SD=SE\times \sqrt{N}$), (2) cholesterol concentrations (total, HDL, and LDL) reported in SI units (mmol/L) were converted to mg/dL (mg/dL = mmol/L×38.67), triglyceride concentrations reported in SI units (mmol/L) were converted to mg/dL (mg/dL = mmol/L×88.57) [24].

The effects of or associations between mushroom consumption and CMD health outcomes were only assessed if three or more articles reported results for a given outcome. Less than three articles reporting results for a given outcome indicate an insufficient amount of evidence for the impact of mushroom consumption. When summarizing the findings, conclusive statements were based on the following criteria: (1) a minimum of 67% of articles need to report the same direction of effect to support a finding, and (2) less than 67% of articles reporting the same direction of effect are considered inconsistent.

## 3. Results

### 3.1. Search Results

**Figure 1:**
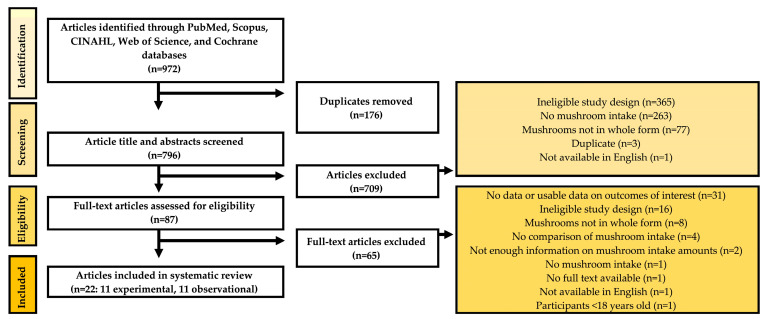
PRISMA flow diagram of the article screening and inclusion process.

Of 972 articles that were identified in our literature search, 176 duplicate articles were removed. A total of 796 articles were independently screened by two researchers, of which 709 were excluded for not meeting inclusion criteria. Of the 87 articles assessed for eligibility in the full-text second-pass screening, 65 were excluded for not meeting inclusion criteria. Ultimately, 22 articles were eligible to be included in this systematic review, as outlined in Figure 1.

### 3.2. Article Characteristics

Of the 22 eligible articles, 11 were experimental, and 11 were observational studies. The qualified experimental research articles included eight placebo-controlled, parallel-design RCTs [25,26,27,28,29,30,31,32], and three non-placebo-controlled (post-intervention vs. baseline) studies [33,34,35]. The sample sizes of the interventional studies ranged from 17 to 1162 participants. Subjects in most experimental studies were generally healthy, with some risk factors of chronic diseases, including dyslipidemia, hypertension, and pre-diabetes. Three studies included clinical participants with HIV or type 2 diabetes mellitus (T2DM). One study included women with pregnancy. The regions of participants included the United States of America, India, Japan, Sri Lanka, Brazil, Germany, and China. The length of study intervention ranged from two weeks to 12 months, and all studies provided only partial study foods (i.e., mushrooms or other select study foods). Three of the experimental studies provided fresh mushrooms, while six provided dried mushrooms, and two did not specify the form. Mushroom types included *Pleurotus ostreatus*, *Pleurotus* spp., *Lentinula edodes*, *Grifola gargal*, and *Agaricus bisporus*. Participants enrolled in interventions serving fresh mushrooms were instructed to consume 100 g daily or 8 ounces thrice weekly. Of the interventions with dried mushrooms, participants were instructed to consume 5–30 g daily. The 11 qualified observational studies included four prospective [36,37,38,39] and six cross-sectional studies [40,41,42,43,44,45]. The eleventh study was a secondary analysis of two different studies, including the Coronary Risk Factors for Atherosclerosis in Women (CORA) Study, which is a case-control study, and the European Prospective Investigation into Cancer and Nutrition (EPIC)-Potsdam Study, which is a prospective study [46]. The sample sizes of the observational studies ranged from 45 to 110,680 participants. Subjects in the observational studies were also generally healthy, with some risk factors of chronic disease, including dyslipidemia. Two studies included clinical populations with T2DM and coronary heart disease. Five of the eleven studies were conducted with participants residing in Japan. Other regions included the United States of America, Korea, Mexico, Italy, and Germany. Among the five prospective studies, the mean follow-up time ranged from 4.6 to 26 years. Although the mushroom type and form were rarely reported in the observational studies, the amounts consumed ranged from 1 to 260 g daily. Mushrooms were generally consumed as part of healthy dietary patterns, described as “prudent”, “vegetable”, or characterized by other generally healthy foods (whole grains, fruits, vegetables, nuts, soy, etc.). All articles included in this systematic review reported protocol approval by their Institutional Review Board. Study characteristics, including the author, year, length of study intervention or follow-up, dietary information, mushroom information (species, form, amount, frequency), sample size, region of participants, health status, age, and BMI, are summarized in Table 3.

### 3.3. Quality Assessment

Of the eleven experimental articles (Table S1), eight were assessed using the NHLBI study quality assessment tool for controlled intervention studies and three using the tool for pre-post studies without a control group. Six of the intervention study articles were rated “poor”, one was rated “fair”, and one was rated “good”. A “poor” rating was assigned to four articles due to lack of intention-to-treat analysis, and other articles received a poor rating due to other “fatal flaws”, such as an overall dropout rate > 20%, lack of randomization, significant differences between groups at baseline, and/or lack of reported study characteristics. The article that received a “fair” rating did not report on differences between groups at baseline and had a differential dropout rate > 15% but included other criteria of the NHLBI study quality assessment tool. The article that was rated “good” did not report on adherence in the treatment group but otherwise met all other criteria of the study quality assessment tool, thus considered to have a low risk of bias. Two articles that were assessed using the pre-post rubric received a “poor” rating due to low participation rate, the insufficient sample size to provide confidence in findings, and unclear prespecified eligibility and selection criteria. The third article received a “fair” rating. While the study did not have a sufficient sample size to detect a change in their primary outcome, and outcomes were not measured more than once at each time point, the study met other criteria of the NHLBI study quality assessment tool, indicating an overall low risk of bias (Table S2).

Of the eleven observational articles (Table S3), seven were rated “poor”, two were rated “fair”, and one was rated “good”. One article was a secondary analysis of two studies, so it was decided to rate the studies separately [46]. Using the NHLBI study quality assessment tool for observational cohort and cross-sectional studies, the prospective cohort study (EPIC) received a “good” rating, and by using the assessment tool for case-control studies, the CORA study received a “fair” rating (Table S4). Articles that received a “poor” rating were mostly cross-sectional studies due to the exposure not being assessed before the outcome, insufficient timeframe, and exposure not being assessed more than once. Articles rated “fair” did not have “fatal flaws”, but they did not assess different levels of exposure and failed to report on whether assessors were blinded. Neither article that received a “good” rating assessed exposure more than once but met other criteria of the study quality assessment tool that are consistent with high internal validity.

### 3.4. Effects of Mushroom Consumption on Cardiometabolic Disease Risk Factors

Our primary outcomes of interest include the effects of and associations between mushroom consumption and diastolic and systolic blood pressures, blood lipids (total cholesterol, HDL cholesterol, LDL cholesterol, triglycerides), fasting plasma glucose, HbA1c, hs-CRP, and morbidity/mortality related to cardiovascular diseases or type 2 diabetes mellitus. Qualitative summaries of the findings, based on the authors’ reporting of statistical significance, are presented in Table 4, Table 5, Table 6 and Table 7. Tables S5–S13 include mean changes and variances (when applicable) or estimated mean changes in the control (when applicable) and intervention groups.

## 4. Systolic and Diastolic Blood Pressures 

Limited evidence from experimental and observational research suggests mushroom consumption has neutral to positive impacts on systolic and diastolic blood pressures.

Among experimental research, three articles reported no influence [28,29,34], and one article reported a reduction [25] in systolic and diastolic blood pressures with greater mushroom consumption.

Another experimental article among pregnant women reported that consumption of 100 g/d *Agaricus bisporus* mushrooms from pre-pregnancy until the 20th gestational week reduced the incidence of gestational hypertension [32].

Among two observational research articles where the objective was to assess the association between mushroom consumption and health, one article reported greater mushroom intake was associated with lower systolic and diastolic blood pressures [44], and one article reported an association with lower diastolic blood pressure only [41].

Among three observational research articles where the objective was to assess the association between high adherence to healthy dietary patterns, including mushrooms and health, two articles reported no association [43,45], and one article reported greater adherence was associated with lower systolic and diastolic blood pressures [40].

## 5. Blood Lipids—Total Cholesterol, HDL Cholesterol, LDL Cholesterol, and Triglycerides

Consistent evidence among experimental research suggests mushroom consumption improves circulating triglyceride concentrations and has a neutral impact on total, HDL, and LDL cholesterol concentrations. Evidence from observational research suggests no association between mushroom consumption and total or LDL cholesterol and mixed findings on HDL cholesterol concentrations and circulating triglycerides (Table 4, Table 5 and Table 6).

### 5.1. Total Cholesterol

Among seven experimental articles, five reported neutral effects [29,30,31,33,34], while two reported a reduction in total cholesterol concentrations [25,28] with greater mushroom consumption.

Similarly, among observational research, six articles reported no association between mushroom consumption and total cholesterol concentrations [37,40,41,43,44,45].

### 5.2. HDL Cholesterol

Among experimental research, six articles reported no effect [28,30,31,33,34,35], and two articles reported an increase (improvement) [25,29] in HDL cholesterol concentrations with greater mushroom consumption.

Among observational research where mushroom consumption was the primary independent variable, one article reported an association between greater mushroom consumption and increased HDL cholesterol concentrations (*p*-trend 0.05) [37]. This observation was only significant when the results of the Nurses’ Health Study and Health Professional Follow-up Study were pooled. No associations were reported in the independent cohorts.

Among observational research where mushrooms were consumed as part of healthy dietary patterns, two articles reported no association [40,43], and one article reported an increase (improvement) [45] in HDL cholesterol concentrations with greater adherence to a healthy dietary pattern, including mushrooms.

### 5.3. LDL Cholesterol

Among experimental research, five articles reported neutral effects [29,30,31,33,34], and two articles reported a reduction in LDL cholesterol concentrations [25,28] with greater mushroom consumption.

One observational research article where mushroom consumption was the independent variable assessed the association between mushroom consumption and LDL cholesterol, of which there was no association reported [37].

Among observational research where mushrooms were consumed as part of healthy dietary patterns, one article reported no association [45] and one article reported an association between greater adherence to a healthy dietary pattern, including mushrooms and reduced LDL cholesterol [40].

### 5.4. Triglycerides

Evidence from six experimental research articles consistently supports a reduction in circulating triglyceride concentrations [25,28,29,30,31,33]. One article reported an increase in triglyceride concentrations after consumption of 100 g/d ultraviolet-treated (500 IU D2/day) mushrooms for 16 weeks [35], but these results were not reproduced in the other three comparison groups.

Of three observational research articles where mushroom consumption was the independent variable, two articles reported no association [37,44], and one article reported a reduction [41] in circulating triglyceride concentrations with greater mushroom consumption.

Among observational research characterized by high adherence to healthy dietary patterns, including mushrooms, one article reported no association [43], and one article reported an association between greater adherence to a healthy dietary pattern, including mushrooms and a reduction [45] in circulating triglycerides.

## 6. Glucose Control—Fasting Plasma Glucose and HbA1c

Evidence from experimental and observational research included in this review does not suggest mushroom consumption influences fasting plasma glucose. While limited experimental research suggests a neutral impact of mushroom consumption on long-term glycemic control (HbA1c), there is insufficient evidence from observational research to support this (Table 4, Table 5 and Table 6).

### 6.1. Fasting Plasma Glucose

Among experimental research, three articles reported no effect [28,31,33], and two articles reported a reduction [25,27] in fasting plasma glucose with greater mushroom consumption. One article reported a reduction in fasting plasma glucose following a six-month weight loss intervention in which mushrooms replaced red meat three days a week [29]. However, this improvement was not sustained during the subsequent six-month weight maintenance phase.

Another experimental article among pregnant women reported greater mushroom consumption through the 20th gestational week reduced the incidence of gestational diabetes compared to the control group [32].

No associations between mushroom consumption and fasting plasma glucose concentrations were reported in observational literature [41,43,44].

### 6.2. HbA1c

Two experimental articles reported no effect [28,35], and one article [25] reported a reduction in long-term glycemic control (HbA1c) with greater mushroom consumption.

One observational article assessed the association between adherence to a healthy dietary pattern, including mushrooms and HbA1c. The authors report no association [43].

## 7. Markers of Inflammation—hs-CRP

Evidence from experimental research suggests greater mushroom consumption may reduce hs-CRP concentrations. There are insufficient results from observational research to draw conclusions appropriately (Table 4, Table 5 and Table 6).

Greater mushroom consumption was reported to reduce hs-CRP concentrations in two [26,29] of three [28] experimental articles.

One observational article assessed the association between mushroom consumption and hs-CRP. The authors reported greater mushroom consumption was associated with higher hs-CRP concentrations (*p*-trend = 0.04) among women from the Nurses’ Health Study. There were no associations between mushroom consumption and hs-CRP among men from the Health Professional Follow-Up Study or in the pooled results [37].

### Associations between Mushroom Consumption and Morbidity/Mortality Related to Cardiovascular Disease or Type 2 Diabetes Mellitus

We also aimed to evaluate the associations between mushroom consumption and morbidity/mortality outcomes related to cardiovascular disease (CVD) and type 2 diabetes mellitus (summarized in Table 7).

## 8. CVD-Related Morbidities and Mortality

For this systematic review, CVD-related morbidities and mortality include cardiovascular disease (CVD), cerebrovascular disease, coronary heart disease (CHD), and stroke. Limited evidence suggests mushroom consumption is not associated with the risk of CVD or CHD morbidity/mortality. There is insufficient evidence for the association between mushroom consumption and the risk of stroke or cerebrovascular disease morbidity/mortality.

### 8.1. Cardiovascular Disease

Among two articles that *a priori* assessed associations between greater mushroom consumption and risks of CVD morbidity or mortality, no associations were reported [36,37].

When mushrooms were consumed as part of a healthy dietary pattern, greater adherence to a healthy dietary pattern, including mushrooms, was associated with a reduced risk of CVD mortality [39].

### 8.2. Cerebrovascular Disease

One article reported greater adherence to a healthy dietary pattern, including mushrooms, was associated with a reduced risk of mortality from cerebrovascular disease [39].

### 8.3. Coronary Heart Disease

Mushroom consumption was not associated with a reduced risk of CHD when mushrooms were the primary independent variable [37].

When mushrooms were consumed as part of healthy dietary patterns, one article reported a reduced risk of CHD among women in a case-control study but not among participants in the European Prospective Investigation into Cancer and Nutrition (EPIC)-Potsdam Study [46]. Another article reported a reduced risk of CHD-cause-specific mortality [39]. Together, these inconsistent results do not suggest an association between mushroom consumption and the risk of CHD morbidity or mortality.

### 8.4. Stroke

One article reported no association between greater mushroom consumption and stroke risk [37].

## 9. T2DM-Related Morbidities and Mortality

T2DM-related morbidities and mortality in this review include hyperglycemia, elevated HbA1c, and T2DM. There is insufficient evidence for the impact of mushroom consumption on the risk of hyperglycemia and elevated HbA1c morbidity. Limited evidence from observational research suggests no association between mushroom consumption and the risk of T2DM morbidity/mortality.

### 9.1. Hyperglycemia

One article assessed the association between mushroom consumption and the risk of hyperglycemia. Among females, the authors report a reduced risk of hyperglycemia when mushrooms were consumed as part of a healthy dietary pattern [38]. However, this association was not observed in males.

### 9.2. Elevated HbA1c

One article reported a reduced odds ratio of having elevated HbA1c with greater adherence to a healthy dietary pattern with mushrooms [42].

### 9.3. Type 2 Diabetes Mellitus (T2DM)

Among three articles, two reported no associations between mushroom consumption and the risk of T2DM morbidity or mortality [36,37], while one reported an increased odds ratio with greater mushroom consumption [44].

## 10. Secondary Outcomes

Our secondary outcomes of interest include very-low-density lipoprotein (VLDL) cholesterol, apolipoprotein A, apolipoprotein B, lipoprotein particle size, fasting insulin, C-peptide, postprandial glucose, and Homeostatic Model Assessment (HOMA). Collectively, there is insufficient evidence to assess the impact of mushroom consumption on any of these outcomes.

### 10.1. Very-Low-Density Lipoprotein (VLDL) Cholesterol

Among experimental research, two articles assessed the effects of mushroom consumption on VLDL cholesterol concentrations. One article reported no effect [33], and one article reported greater mushroom consumption reduced VLDL cholesterol concentrations [25].

### 10.2. Apolipoprotein A

This outcome was not assessed by any articles in the current review.

### 10.3. Apolipoprotein B

This outcome was not assessed by any articles in the current review.

### 10.4. Lipoprotein Particle Size

This outcome was not assessed by any articles in the current review.

### 10.5. Fasting Insulin

One experimental article reported consuming a Japanese diet with higher mushroom content, compared to a partial Japanese diet with lower mushroom content, led to a greater reduction in fasting insulin concentrations (differential −1.2 (−2.3, 0.0) μU/mL, *p* = 0.033) [28].

### 10.6. C-Peptide

One observational article reported no association between greater mushroom consumption and C-peptide [37].

### 10.7. Postprandial Glucose

Among two experimental articles, one article reported no effect [35], and one article reported a reduction [27] in postprandial glucose concentrations with greater mushroom consumption.

### 10.8. Homeostatic Model Assessment (HOMA)

One experimental article reported no effect of mushroom consumption on HOMA [35].

## 11. Discussion

To our knowledge, this is the most comprehensive systematic review assessing the impact of mushroom consumption on CMD health conducted to date. Evidence from experimental research suggests mushroom consumption improves serum/plasma triglycerides and hs-CRP, but not other lipids, lipoproteins, measures of glucose control (fasting glucose and HbA1c), or blood pressure. Evidence from observational research suggests no association between mushroom consumption and fasting total or LDL cholesterol, glucose, or morbidity/mortality from CVD, CHD, or T2DM. Inconsistent or insufficient findings were reported for other CMD health outcomes in observational literature. Our review differs from previous ones in that we are assessing the effects of and associations between all mushroom species on CMD risk factors and morbidities/mortality using evidence from both experimental and observational research. Previous work has examined the effects of *Pleurotus ostreatus* (oyster) [20] or *Agaricus bisporus* (white button, crimini, or portabella) [19] only. The third systematic review we are aware of did not specify the mushroom species but stated the review included only observational research [21]. We found an insufficient number of articles to complete a sub-analysis on the effects of consuming different mushroom species on CMD health outcomes. It is also noteworthy our review includes whole mushrooms only in fresh or dried forms, whereas previous reviews included studies with interventions using bioactive extracts derived from mushrooms (i.e., polysaccharides) [19,21], which may be in greater concentrations than occur naturally in dietary mushrooms. Given the differences between our review and previous ones, a comparison of results should be made cautiously. Since our review includes all mushroom species combined, our conclusion applies broadly to mushrooms as food, not to a specific mushroom species.

Despite many articles in this review reporting at least one beneficial effect of or association between mushroom consumption and CMD health, the strength of evidence is weak, in part due to the lack of robust experimental and observational research. As previously described, most experimental research articles were rated “poor” using the NHLBI study quality assessment tool. Strengths among controlled intervention studies included randomization of participants, concealed treatment allocation, valid assessment of outcomes, and prespecified outcomes prior to analysis. However, “fatal flaws”, which seriously increase the risk of bias, included an overall dropout rate of >20% in two articles, a high differential dropout rate between groups (i.e., >15%) in three articles (not reported in two), and no intention-to-treat analysis in four articles (not reported in two). Having a “fatal flaw” automatically deemed the study as having a high risk of bias, and thus, it was rated “poor” quality. Strengths of the pre-post experimental studies included defined objectives, clear description and consistent delivery of the intervention, valid outcome measures, and statistical tests to examine pre vs. post changes. None of the pre-post experimental studies assessed outcomes multiple times before and after the intervention or reported a sufficient sample size to provide confidence in findings. The risk of bias was high (i.e., rated “poor”) in two articles due to lack of (or lack of reporting on) a representative sample of the population of interest, enrollment of all participants meeting inclusion criteria, and use of blinded assessors. Another limitation of the experimental research broadly is the lack of any full-feed RCTs assessing the effect of mushroom consumption on CMD health. Without controlling dietary intake, it is unclear whether mushroom consumption alone influences health. Finally, interventions, including mushroom consumption, were highly variable across articles, making it difficult to create generalizations or recommendations about the species, form (fresh or dried), amount, or duration needed to impact health. Recommendations for future research include the use of fully controlled dietary interventions and a minimum daily intake of one cup of equivalent fresh or dried mushrooms. A one-cup equivalent minimum daily intake would allow for easier comparisons between study interventions and consistent messaging to consumers. In summary, while we have information on mushrooms and CMD health, the limitations described herein contribute to the lack of suitable data for a meta-analysis. There is a need and opportunity for future research, with the considerations described here, to help move the field forward and provide suitable data to be included in future meta-analyses.

Regarding observational research, the strengths of cohort and cross-sectional studies included defined objectives, clearly specified populations of interest, recruitment of subjects from similar populations, and the use of valid outcome measures. The high risk of bias among many articles was attributed to a lack of measuring the exposure before the outcome, sufficient timeframe, examining different levels of exposure (i.e., assessing a dose-response relationship), and assessing the exposure more than once over time. Most observational research also failed to report the sample justification and whether outcome assessors were blinded. Another limitation of the observational literature is the lack of an *a priori* assessment of the associations between mushroom consumption and CMD health. Among the 11 articles included in this review, only four articles *a priori* sought to examine the associations between mushroom consumption and some parameter of cardiometabolic health. The remaining seven articles assessed the associations between high adherence to healthy dietary patterns, including mushrooms and cardiometabolic health. Given these dietary patterns were characterized by other healthful foods, we can’t confidently say that health impacts are associated with mushroom consumption, but rather consuming a healthy dietary pattern that includes mushrooms. There was also insufficient information about the mushroom species, form, quantity, or preparation methods used by participants. With these limitations in mind, our recommendations for future observational research include the design of *a priori* research examining the associations between mushroom consumption and cardiometabolic health, assessment of mushroom consumption at more than one time and different levels of exposure (i.e., dose–response relationship), and better documentation of the mushrooms (i.e., species, form, quantity, preparation, etc.) consumed by participants. In sum, the limitations of the literature warrant serious caution when interpreting the findings and comparing the consistency of evidence between experimental and observational research.

While there is weak evidence in human research to support a beneficial impact of mushroom consumption on cardiometabolic health, this is likely attributable to weaknesses in the study designs described above and not because mushrooms do not promote health. Importantly, the availability of experimental and observational research in humans does not include critical assessments by which bioactive compounds in mushrooms may influence health. However, as described in the introduction, mushrooms contain several bioactive compounds, including beta-glucans, lovastatin, L-ergothioneine, ergosterol, and polyphenols, among others, which are known to have health benefits [47,48]. The insufficient evidence presented in this review is not a reason to abandon this line of research but rather an opportunity to improve the design of future studies to better inform this body of literature and highlight areas for further exploration.

A strength of this work is that it is the most comprehensive review on this topic. The use of a systematic literature search strategy developed for five databases by an experienced health and life sciences librarian gives us confidence that we have exhaustively searched the literature to address this research question for humans. The inclusion of both RCTs and observational research has allowed us to examine the impact of mushroom consumption on CMD health outcomes broadly and is not limited to specific mushroom species. Since we are looking at mushrooms from a dietary rather than a pharmaceutical perspective, our findings represent the impacts that would likely be seen in the general population (i.e., not at pharmacological doses or in acute clinical populations). Finally, our review meets the Preferred Reporting Items for Systematic review and Meta-Analysis Protocols (PRISMA-P) 2015 checklist guidelines. Our review is limited by the available literature on this topic and by many articles that reported inadequate details on mushroom consumption and/or health outcomes. Articles that did not have a comparison of higher vs. lower mushroom consumption were not included in this review.

## 12. Conclusions

Mushroom consumption improves serum/plasma triglycerides and hs-CRP, as supported by limited evidence from experimental research. Neutral impacts were reported for other lipids, lipoproteins, measures of glucose control (fasting glucose and HbA1c), and blood pressure. Limited evidence from observational research suggests no association between mushroom consumption and fasting blood total or LDL cholesterol, glucose, or morbidity/mortality from CVD, CHD, or T2DM. Inconsistent or insufficient findings were reported for other CMD health outcomes. The quality of most articles included in this review raised concerns due to study methodology and/or poor reporting issues. While new, high-quality experimental and observational research is warranted, limited experimental findings suggest greater mushroom consumption lowers blood triglycerides and hs-CRP, indices of cardiometabolic health.

## Figures and Tables

**Table 1:** Description of the research question and PICOS for a systematically searched literature review.

Parameter	Description
Population	Adult humans (Age ≥ 18 y)
Intervention	Groups consuming mushrooms or statistically significantly higher amounts of mushrooms
Comparison	Groups not consuming mushrooms or groups consuming statistically significantly lower amounts of mushrooms
Outcomes	Cardiometabolic disease risk factors and morbidities. Primary: diastolic and systolic blood pressures, blood lipids (total cholesterol, HDL cholesterol, LDL cholesterol, triglycerides), fasting plasma glucose, HbA1c, hs-CRP, and morbidity/mortality related to cardiovascular diseases or type 2 diabetes mellitus Secondary: other lipoproteins (VLDL, apolipoprotein A, apolipoprotein B), lipoprotein particle size, fasting insulin, C-peptide, postprandial glucose
Study Design	Peer-reviewed randomized controlled trials and observational studies
Research Question	In adults, what is the effect of mushroom consumption on cardiometabolic disease risk factors and morbidities compared to those not consuming mushrooms?

**Table 2:** Search terms and results for a systematically searched literature review.

Source	Search Terms	Results
PubMed	(Mushroom * OR Agaricales [MeSH Terms] OR Shiitake Mushrooms [MeSH Terms] OR Pleurotus [MeSH Terms] OR Grifola [MeSH Terms]) AND (blood pressure OR blood pressure [MeSH Terms] OR triglycerides OR triglycerides [MeSH Terms] OR Cholesterol OR Epicholesterol OR cholesterol [MeSH Terms] OR cholesterol, LDL [MeSH Terms] OR LDL Cholesterol OR Low Density Lipoprotein Cholesterol OR cholesterol, HDL [MeSH Terms] OR High-Density Lipoprotein Cholesterol OR HDL Cholesterol OR blood glucose OR blood glucose [MeSH Terms] OR blood sugar OR diabetes OR Diabetes Mellitus [MeSH Terms] OR hba1c OR Glycated Hemoglobin A OR Glycated Hemoglobin A [MeSH Terms] OR hs-CRP OR High Sensitivity C-Reactive Protein OR C-Reactive Protein [MeSH Terms] OR CRP OR cardiovascular diseases [MeSH Terms] OR cardiovascular disease * or heart disease * OR vascular disease * OR “diabetes mellitus, type 2” [MeSH Terms] OR diabetes type 2 OR type 2 diabetes) AND (Humans [MeSH Terms])	570
CINAHL	(Mushroom * OR Agaricales OR Shiitake Mushrooms OR Pleurotus OR Grifola) AND (blood pressure OR triglycerides OR Cholesterol OR Epicholesterol OR LDL Cholesterol OR Low Density Lipoprotein Cholesterol OR High-Density Lipoprotein Cholesterol OR HDL Cholesterol OR blood glucose OR blood sugar OR diabetes OR Diabetes Mellitus OR hba1c OR Glycated Hemoglobin A OR hs-CRP OR High Sensitivity C-Reactive Protein OR CRP OR type 2 diabetes OR cardiovascular disease) AND (Humans)	76
Scopus	Mushroom * AND (“blood pressure” OR Cholesterol OR “blood glucose” OR “blood sugar” OR diabetes OR “Diabetes Mellitus” OR “type 2 diabetes” OR “cardiovascular disease”) AND Humans	69
Web of Science	(Mushroom * OR Agaricales OR Shiitake Mushrooms OR Pleurotus OR Grifola) AND (blood pressure OR triglycerides OR Cholesterol OR Epicholesterol OR LDL Cholesterol OR Low Density Lipoprotein Cholesterol OR High-Density Lipoprotein Cholesterol OR HDL Cholesterol OR blood glucose OR blood sugar OR diabetes OR Diabetes Mellitus OR hba1c OR Glycated Hemoglobin A OR hs-CRP OR High Sensitivity C-Reactive Protein OR CRP OR type 2 diabetes OR cardiovascular disease) AND (Humans)	256
Cochrane Library	(Mushroom * OR Agaricales OR Shiitake Mushrooms OR Pleurotus OR Grifola) AND (blood pressure OR triglycerides OR Cholesterol OR blood glucose OR diabetes OR Glycated hemoglobin A OR C-Reactive Protein OR type 2 diabetes OR cardiovascular disease)	1

* An asterisk indicates a truncated search term.

**Table 3:** Description of study characteristics.

Author, Year	Study Type and Design	Length of Study Intervention or Follow-Up	Dietary Description	Mushroom Type	Mushroom Form	Mushroom Amount and Frequency	Sample Size	Region	Healthy or Diseased	Age (Years) †	BMI (kg/m^2^) †
Abrams et al., 2011 [33]	Exp, Single-arm	8 weeks	Partial feed	*Pleurotus ostreatus*	Dried	15 g daily	20	USA	HIV	36–60	NR
Agrawal et al., 2010 [25]	RCT, Parallel	3 months	Partial feed	*Pleurotus* spp.	NR	NR	111	India	T2DM	51.1 ± 8.3 *	26.67 ± 4.5 *
Dai et al., 2015 [26]	RCT, Parallel	4 weeks	Partial feed	*Lentinula edodes*	Dried	5 or 10 g daily	52	USA	Healthy	21–41 *	M: 23.3 ± 7.2 * F: 22.4 ± 8.8 *
Harada et al., 2016 [34]	Exp, Single-arm	2 weeks	Partial feed	*Grifola gargal*	Dried	5 g daily	17	Japan	NR	61.2 ± 7.6	NR
Jayasuriya et al., 2015 [27]	RCT, Parallel	2 weeks	Partial feed	*P. ostreatus* and *P. cystidiousus*	Dried	50 mg/kg BW daily	88	Sri Lanka	Healthy	NR	NR
Maruyama et al., 2021 [28]	RCT, Parallel	6 months	Partial feed	NR	NR	40 ± 33 g daily	98	Japan	Dys-lipidemia, T2DM, HTN	53.5 ± 8.2 *	24.4 ± 3.7 *
Mehrotra et al., 2014 [35]	RCT, Parallel (pre vs. post)	16 weeks	Partial feed	NR	Fresh	100 g daily	36	USA	Pre-diabetic	49 ± 12	NR
Poddar et al., 2013 [29]	RCT, Parallel	12 months	Partial feed	*Agaricus bisporus*	Fresh	8 oz, 3x/week	73	USA	Healthy	48.4 ± 12	25–40
Schneider et al., 2011 [30]	RCT, Parallel	21 days	Partial feed	*Pleurotus ostreatus*	Dried	30 g daily	20	Germany	Hyper-lipidemia	20–34	22.7 ± 3.7 *
Spim et al., 2021 [31]	RCT, Parallel	66 days	Partial feed	*Lentinula edodes*	Dried	3.5 g daily	68	Brazil	Dys-lipidemia	40 ± 11	26.9 ± 4.4
Sun and Niu, 2020 [32]	RCT, Parallel	Pre-pregnancy-20th week gestation	Partial feed	*Agaricus bisporus*	Fresh	100 g daily	1162	China	Healthy, pregnant	31.2 ± 4.5 *	22.47 ± 3.66 *
Ba et al., 2021 [36]	OBS, Pro-spective	19.5 ± 7.4-year follow-up	NR	NR	NR	10–72 g daily	15,546	USA	NR	44.3 ± 0.5	NR; ~45% with BMI <24.9
Htun et al., 2018 [40]	OBS. Cross-sectional	NA	Traditional Japanese	NR	NR	NR; loading factor 0.35	8721	Japan	NR	40–74	M: 24.3 ± 3.0 * F: 23.1 ± 3.4 *
Lee DH et al., 2019 [37]	OBS, Pro-spective	26 year follow-up	Prudent	NR	Fresh, cooked, canned	5 servings per week	110,680	USA	Healthy	M: 53.2 ± 9.2 * F: 52.3 ± 6.9 *	M: 25.7 ± 3.6 * F: 25.2 ± 4.6 *
Lee KW et al., 2019 [38]	OBS, Pro-spective	4.9 year follow-up	Prudent	NR	NR	NR; loading factor 0.55 (M), 0.56 (F)	55,457	Korea	Healthy	40–79	M: 24.5 ± 2.6 F: 23.6 ± 2.8
Meneses et al., 2020 [41]	OBS, Cross-sectional	NA	Traditional Oaxaca Foods	Wild and cultivated mushrooms	Fresh, cooked	260 g daily	45	Mexico	Dys-lipidemia	48.27 ± 14.08	28.69 ± 4.56
Nanri et al., 2017 [39]	OBS, Pro-spective	5- and 10-year follow-up	Prudent	NR	NR	5–16 g daily	81,720	Japan	Healthy	40–69	23.5 ± 0.2
Okada et al., 2019 [42]	OBS, Cross-sectional	NA	Vegetable	NR	NR	6.47 ± 12.7 to 40.3 ± 45.8 g daily	9550	Japan	Healthy	64.4 ± 10.8 *	23.2 ± 3.3 *
Osonoi et al., 2016 [43]	OBS, Cross- sectional	NA	Seaweed, veg, soy, mushrooms	NR	NR	NR; loading factor 0.55	726	Japan	T2DM	57.8 ± 8.6	24.6 ± 4.1
Pounis et al., 2013 [44]	OBS, Cross-sectional	NA	Grains, nuts/ seeds, legumes, poultry, fish	NR	NR	<14, 14–28, or >28 g/week	13,770	Italy	Healthy	53.1 ± 11.0	NR; Obesity prevalence 25.2–28.8%
Uchiyama et al., 2022 [45]	OBS, Cross-sectional	NA	Traditional Japanese	NR	NR	0.20 (0.18–0.40) to 0.40 (0.20-0.80) •	198	Japan	Healthy	37 (28–44) ^‡^	21.2 (19.8–23) ‡
Weikert et al., 2005 [46]	OBS, Case-control (CORA)	NA	Whole-grain bread, fresh fruit, olive oil, mushrooms, cruciferous vegetables, wine, and nuts	NR	NR	1.0–4.0 g daily	455	Germany	Coronary Heart Disease	30–80	26.1 ± 4.8 (case) 25.6 ± 4.3 (con)
	OBS, Pro-spective (EPIC)	4.6 year follow-up		NR	NR	1.0–3.5 g daily	26,795		Healthy	35–65	27.5 ± 3.8 (case) 26.3 ± 4.4 (con)

* Data for mushroom group only. † Data presented as Mean ± SD or range. ‡ Data presented as Median (IQR). • Median (IQR) in grams per ideal body weight. Abbreviations: BMI: body mass index; Exp: experimental; g: grams; USA: United States of America; NR: not reported; RCT: randomized controlled trial; T2DM: type 2 diabetes mellitus; HTN: hypertension; BW: body weight; M: male; F: female; OBS; observational; NA: not applicable; veg: vegetable; Q1: quartile 1; Q4: quartile 4; con: control.

**Table 4:** Summary of experimental and observational studies reporting on primary outcomes.

Parameter	Experimental Research				Observational Research			
		IMPACT †				IMPACT		
	Total # of Articles	+	↔	−	Total # of Articles	+	↔	−
Systolic Blood Pressure	5	40 (2) *	60 (3)	0 (0)	5	40 (2) #	60 (3)	0 (0)
Diastolic Blood Pressure	5	40 (2) *	60 (3)	0 (0)	5	60 (3) #	40 (2)	0 (0)
Total Cholesterol	7	29 (2)	71 (5)	0 (0)	6	0 (0)	100 (6) #	0 (0)
HDL Cholesterol	8	25 (2)	75 (6)	0 (0)	4	50 (2)	50 (2)	20 (1)
LDL Cholesterol	7	29 (2)	71 (5)	0 (0)	3	33 (1)	67 (2)	0 (0)
Triglycerides	7	86 (6)	0 (0)	14 (1)	5	40 (2)	60 (3) #	0 (0)
Glucose	7	57 (4) *	43 (3)	0 (0)	3	0 (0)	100 (3)	0 (0)
HbA1c	3	33 (1)	67 (2)	0 (0)	1	0 (0)	100 (1)	0 (0)
hs-CRP	3	67 (2)	33 (1)	0 (0)	1	0 (0)	100 (1)	0 (0)

† Results are displayed based on statistically significant differences in the original manuscript. Results displayed as percent (number) of studies reporting a positive, neutral, or negative impact of mushroom consumption on the outcome in the intervention group. # Results from Pounis et al. (2013) are based on the percentage of participants with hypertension, hypercholesterolemia, and hypertriglyceridemia, listed under Systolic Blood Pressure/Diastolic Blood Pressure, Total Cholesterol, and Triglycerides, respectively. * Results from Sun and Niu (2020) on gestational hypertension and gestational diabetes are listed under Systolic Blood Pressure/Diastolic Blood Pressure and Glucose, respectively.

**Table 5:** Qualitative summary of experimental studies evaluating cardiometabolic disease risk factors in adults consuming higher vs. lower amounts of mushrooms.

*Author*, *Year*	*Diet Group*	*Comparator*	*Mushroom Species and Form*	*Sys-BP* *†*	*Dia-BP*	*TC*	*HDL*	*LDL*	*TAG*	*Glu*	*HbA1c*	*hs-CRP*
***Abrams et al., 2011*** [33]	Post, 15 g daily	Baseline	*Pleurotus ostreatus*, dried	**NR**	**NR**	**≠**	**≠**	**≠**	**↓**	**≠**	**NR**	**NR**
***Agrawal et al., 2010*** [25]	Mushroom biscuits	Baseline	*Pleurotus* spp., NR	**↓**	**↓**	**↓**	**↑**	**↓**	**↓**	**↓**	**↓**	**NR**
	Mushroom biscuits	Ajwain biscuits	*Pleurotus* spp., NR	**↓**	**↓**	**↓**	**↑**	**↓**	**↓**	**↓**	**↓**	**NR**
***Dai et al., 2015*** [26]	5 g and 10 g/daily •	Baseline	*Letinula edodes*, dried	**NR**	**NR**	**NR**	**NR**	**NR**	**NR**	**NR**	**NR**	**↓**
***Harada et al., 2016*** [34]	Post, 5 g/daily	Baseline	*Grifola gargal*, dried	**≠**	**≠**	**≠**	**≠**	**≠**	**NR**	**NR**	**NR**	**NR**
***Jayasuriya et al., 2015*** [27]	*P. ostreatus*, 50 mg/kg/bw daily	Control (water)	*Pleurotus ostreatus*, dried	**NR**	**NR**	**NR**	**NR**	**NR**	**NR**	**↓**	**NR**	**NR**
	*P. cystidiousus*, 50 mg/kg/bw daily	Control (water)	*Pleurotus cystidiousus*, dried	**NR**	**NR**	**NR**	**NR**	**NR**	**NR**	**↓**	**NR**	**NR**
***Maruyama et al., 2021*** [28]	Japanese Diet, 40 ± 33 g/d at 6 months	Partial Japanese Diet, 31 ± 27 g/d at 6 months	NR	**≠**	**≠**	**↓**	**≠**	**↓**	**↓**	**≠**	**≠**	**≠**
***Mehrotra et al., 2014*** [35]	Ultraviolet treated mushrooms (500 IU D_2_/day) + placebo, 100 g/daily	Baseline	NR, fresh	**NR**	**NR**	**NR**	**≠**	**NR**	**↑**	**NR**	**≠**	**NR**
	Ultraviolet treated mushrooms (2600 IU D_2_/day) + placebo, 100 g/daily	Baseline	NR, fresh	**NR**	**NR**	**NR**	**≠**	**NR**	**≠**	**NR**	**≠**	**NR**
	Untreated mushrooms + 1200 IU D_3_/day capsules, 100 g/daily	Baseline	NR, fresh	**NR**	**NR**	**NR**	**≠**	**NR**	**≠**	**NR**	**≠**	**NR**
	Untreated mushrooms + 7300 IU D_3_/day capsules, 100 g/daily	Baseline	NR, fresh	**NR**	**NR**	**NR**	**≠**	**NR**	**≠**	**NR**	**≠**	**NR**
***Poddar et al., 2013*** ¶ [29]	Mushroom diet, 8 oz on 3 d/wk, 0–6 months WL	Meat diet, 90% lean ground beef 3 d/wk, 0–6 months WL	Agaricus bisporus, fresh	**≠**	**≠**	**≠**	**NR**	**≠**	**NR**	**NR**	**NR**	**↓**
	Mushroom diet, 8oz on 3 d/wk, 6–12 months WM	Meat diet, 90% lean ground beef 3 d/wk, 6–12 months WM	*Agaricus bisporus*, fresh	**NR**	**NR**	**≠**	**NR**	**≠**	**NR**	**NR**	**NR**	**NR**
	Mushroom diet, 8oz on 3 d/wk, 0–6 months WL	Baseline	*Agaricus bisporus*, fresh	**NR**	**NR**	**NR**	**≠**	**NR**	**↓**	**↓**	**NR**	**NR**
	Mushroom diet, 8 oz on 3 d/wk, 12 months	Baseline	*Agaricus bisporus*, fresh	**NR**	**NR**	**NR**	**↓**	**NR**	**↓**	**≠**	**NR**	**NR**
***Schneider et al., 2011*** [30]	Verum diet, 30 g/d	Baseline	*Pleurotus ostreatus*, freeze-dried	**NR**	**NR**	**≠**	**≠**	**≠**	**↓**	**NR**	**NR**	**NR**
***Spim et al., 2021*** [31]	Intervention group, 3.5 g/d	Placebo group	*Letinula edodes*, dried	**NR**	**NR**	**≠**	**≠**	**≠**	**↓**	**≠**	**NR**	**NR**
***Sun and Niu, 2020 §*** [32]	MD group, 100 g/d	Placebo group	*Agaricus bisporus*, fresh	**↓**	**↓**	**NR**	**NR**	**NR**	**NR**	**↓**	**NR**	**NR**

† Results are displayed based on statistically significant differences in the original manuscript. ↑: increase; ↓: decrease; ≠: no change; NR: not reported/not evaluated. • The authors indicated that data from both groups (5 g/daily and 10 g/daily) were combined for analysis. **¶** Indicates that the study was designed with a weight loss intervention. § Results on gestational hypertension and gestational diabetes are listed under Sys-BP/Dia-BP and Glucose, respectively. Abbreviations: Sys-BP: systolic blood pressure; Dia-BP: diastolic blood pressure; TC: total cholesterol, HDL: high-density lipoprotein cholesterol; LDL: low-density lipoprotein cholesterol; TAG: triglycerides; Glu: glucose; HbA1c: hemoglobin A1c; hs-CRP: high-sensitivity C-reactive protein; g: grams; NR: not reported; mg: milligrams; kg: kilograms; bw: body weight; g/d: grams per day; IU; international units; d/wk: days per week; WL: weight loss; WM: weight maintenance.

**Table 6:** Qualitative summary of observational studies evaluating cardiometabolic disease risk factors in adults consuming higher vs. lower amounts of mushrooms.

*Author,* *Year*	*Diet Group*	*Comparator*	*Mushroom Species and Form*	*Sys-BP* †	*Dia-BP*	*TC*	*HDL*	*LDL*	*TAG*	*Glu*	*HbA1c*	*hs-CRP*
Lee DH et al., 2019 *‡ [37]	5 servings/wk (pooled)	Never (pooled)	NS, fresh, cooked, canned	**NR**	**NR**	**≠**	**↑**	**≠**	**≠**	**NR**	**NR**	**≠**
Meneses et al., 2020 * [41]	High frequency, daily consumption	No consumption	Wild and cultivated mushrooms	**≠**	**↓**	**≠**	**NR**	**NR**	**↓**	**≠**	**NR**	**NR**
Pounis et al., 2013 *# [44]	Tertile 3, >28 g/wk	Tertile 1, <14 g/wk	NS	**↓**	**↓**	**≠**	**NR**	**NR**	**≠**	**≠**	**NR**	**NR**
***Htun et al., 2018*** [40]	Traditional Japanese, Q4	Traditional Japanese Q1	NS	**↓**	**↓**	**≠**	**≠**	**↓**	**NR**	**NR**	**NR**	**NR**
***Osonoi et al., 2016*** [43]	Seaweed, vegetable, soy products, and mushroom diet, Q5	Seaweed, vegetable, soy products, and mushroom diet, Q1	NS	**≠**	**≠**	**≠**	**≠**	**NR**	**≠**	**≠**	**≠**	**NR**
***Uchiyama et al., 2022*** [45]	Traditional Japanese T3, 0.40 (0.20–0.80) g ^	Traditional Japanese T1, 0.20 (0.18–0.40) g ^	NS	**≠**	**≠**	**≠**	**↑**	**≠**	**↓**	**NR**	**NR**	**NR**

† Results are displayed based on statistically significant differences in the original manuscript. ↑: increase; ↓: decrease; ≠: no change; NR: not reported or not evaluated. * *A priori* assessment of the associations between mushroom consumption and cardiometabolic health. ‡ The authors report an improvement in HDL from the pooled analysis only (*p*-trend = 0.05). Improvements were not reported for the independent cohorts (Nurses’ Health Study and Health Professionals’ Follow-up Study). Mushroom consumption was reported to be associated with an increase in hs-CRP in women from the Nurses’ Health Study only. No associations were reported between mushroom consumption and hs-CRP in the men from the Health Professionals Follow-up Study or when the results of the two cohorts were pooled. # Results are based on the percentage of participants with hypertension, hypercholesterolemia, and hypertriglyceridemia, listed under Sys-BP/Dia-BP, TC, and TAG, respectively. ^ Median (IQR) in grams per ideal body weight.

**Table 7:** Qualitative summary of studies evaluating cardiometabolic disease morbidities and mortality in adults.

*Author,* *Year*	*Diet Group*	*Comparator*	*Mushroom Type (Fresh, Dried, Species)*	*CVD* †	*Cerebrovascular Disease*	*CHD*	*Stroke*	*T2DM*	*Hyper-Glycemia*	*Elevated* *HbA1c*
***Ba et al., 2021 ^***‡ [36]	Mushroom intake, 10–72 g/d	No mushroom intake	NR	HR: 0.82 (0.56, 1.21)	NR	NR	NR	HR: 0.32 (0.06, 1.65)	NR	NR
***Lee DH et al., 2019 ^*** [37]	5 servings/wk, female	Never, female	NR, fresh, cooked, canned	HR: 1.08 (0.94, 1.25)	NR	HR: 1.09 (0.90, 1.32)	HR: 1.08 (0.86, 1.34)	HR: 1.04 (0.91, 1.19)	NR	NR
	5 servings/wk, male	Never, male		HR: 0.93 (0.78, 1.11)	NR	HR: 0.89 (0.72, 1.10)	HR: 1.04 (0.75, 1.43)	HR: 1.04 (0.83, 1.31)	NR	NR
	5 servings/wk, pooled	Never, pooled		HR: 1.02 (0.91, 1.14)	NR	HR: 1.00 (0.87, 1.16)	HR: 1.05 (0.87, 1.25)	HR: 1.04 (0.93, 1.16)	NR	NR
***Pounis et al., 2013 ^*** [44]	Tertile 3, male, >28 g/wk	Tertile 1 male, <14 g/wk	NR	NR	NR	NR	NR	OR: 1.27 (1.05, 1.55) *	NR	NR
	Tertile 3, female, >28 g/wk	Tertile 1, female, <14 g/wk	NR	NR	NR	NR	NR	OR: 1.38 (1.05, 1.81) *	NR	NR
***Lee KW et al., 2019*** [38]	Prudent Q5, male	Prudent Q1, male	NR	NR	NR	NR	NR	NR	HR: 0.93 (0.75, 1.15)	NR
	Prudent Q5, female	Prudent Q1, female	NR	NR	NR	NR	NR	NR	HR: 0.75 (0.63, 0.89) *	NR
***Nanri et al., 2017***‡ [39]	Prudent Q4, 16 g/d	Prudent Q1, 5 g/d	NR	HR: 0.72 (0.64, 0.79) *	HR: 0.63 (0.53, 0.75) *	HR: 0.75 (0.66, 0.87) *	NR	NR	NR	NR
***Okada et al., 2019*** [42]	Vegetable Q4, 40.3 ± 45.8 g/d	Vegetable Q1, 6.47 ± 12.7 g/d	NR	NR	NR	NR	NR	NR	NR	OR: 0.68 (0.49, 0.95) *
***Weikert et al., 2005*** [46]	CORA Q5, 4.0 ± 0.4 g/d	CORA Q1, 1.0 ± 0.1 g/d	NR	NR	NR	RR: 0.39 (0.17, 0.92) *	NR	NR	NR	NR
	EPIC Q5, 3.5 ± 0.1 g/d	EPIC Q1, 1.0 ± 0.1 g/d	NR	NR	NR	RR: 0.72 (0.43, 1.20)	NR	NR	NR	NR

^ *A priori* assessment of the associations between mushroom consumption and cardiometabolic health. † Results are displayed as Risk Ratio (RR) (95% Confidence Interval), Hazard Ratio (HR) (95% Confidence Interval), or Odds Ratio (OR) (95% Confidence Interval). ‡ Results are displayed as Hazard Ratio (HR) (95% Confidence Interval) of cause-specific mortality. * An asterisk indicates statistical significance, *p* <0.05. Abbreviations: CVD: cardiovascular disease; CHD: coronary heart disease; T2DM: type 2 diabetes mellitus; NR: not reported or not evaluated.

## Data Availability

The data presented in this study are available in the article and Supplementary Material.

## Supplementary Materials

The following supporting information can be downloaded at: https://www.mdpi.com/article/10.3390/nu15051079/s1, Table S1: NHLBI Study Quality Assessment Tool for Controlled Intervention Studies, Table S2: NHLBI Study Quality Assessment Tool for Pre-Post Studies Without a Control group, Table S3: NHLBI Study Quality Assessment Tool for Observational Cohort and Cross-sectional Studies, Table S4: NHLBI Study Quality Assessment Tool for Case-Control Studies, Table S5: Change in Systolic Blood Pressures (mmHg), Table S6: Change in Diastolic Blood Pressures (mmHg), Table S7: Change in Total Cholesterol (mg/dL), Table S8: Change in HDL Cholesterol (mg/dL), Table S9: Change in LDL Cholesterol (mg/dL), Table S10: Change in Triglycerides (mg/dL), Table S11: Change in Fasting Glucose (mg/dL), Table S12. Change in HbA1c (%), Table S13: Change in hs-CRP (mg/dL).
