# Supplementary material for: An Assessment of Mushroom Consumption on Cardiometabolic Disease Risk Factors and Morbidities in Humans: A Systematic Review
Source: Nutrients. 2023 Feb 21;15(5):1079. doi: 10.3390/nu15051079 (PMC10005148; doi:10.3390/nu15051079)
Supplement: Supplementary file 1 [file nutrients-15-01079-s001.zip › nutrients-2193586-supplementary.pdf]

Table S1. NHLBI Study Quality Assessment Tool for Controlled Intervention Studies.

	Randomization	Adequate method of randomization	Concealed treatment allocation	Blinded participants and providers	Blinded outcomes assessors	Groups similar at baseline	Overall dropout rate ≤20%	Differential dropout rate ≤15%	High adherence to intervention	Other interventions avoided or similar	Valid, reliable, and consistent assessment of outcomes	Sufficiently large sample size *	Prespecified outcomes prior to analysis	Intention-to-treat analysis	Overall Rating
**Agrawal et al., 2010**	√	?	√	√	√	X	√	X	?	√	√	?	√	X	**Poor**
**Dai et al., 2015**	√	√	√	?	?	?	√	X	√	√	√	√	√	√	**Fair**
**Jayasuriya et al., 2015**	√	X	√	X	?	?	?	?	?	?	√	?	√	?	**Poor**
**Maruyama et al., 2021**	√	√	√	√	√	√	√	√	?	√	√	√	√	√	**Good**
**Poddar et al., 2013**	√	?	?	X	√	√	X	?	X	√	√	?	√	X	**Poor**
**Spim et al., 2021**	√	√	√	√	√	√	X	X	?	√	√	?	√	?	**Poor**
**Schneider et al., 2011**	√	X	?	?	?	√	√	√	?	√	√	?	√	X	**Poor**
**Sun and Niu, 2020**	√	√	√	X	?	√	√	√	√	√	√	√	√	X	**Poor**

* A sufficiently large sample size had to be able to detect a difference in the main outcome between groups with at least >80% power. √ **- Yes.** X **– No.** ? **– Not reported by the authors in the original manuscript**.

Table S2. NHLBI Study Quality Assessment Tool for Pre-Post Studies Without a Control Group.

	Objective study question	Clearly prespecified eligibility and selection criteria	Participants are representative of the population of interest	All participants meeting entry criteria enrolled	Sample size large enough to provide confidence in findings	Clearly described and consistently delivered intervention	Prespecified, defined, valid, reliable, consistent outcome measures	Blinded assessors	Loss to follow-up ≤20%, loss accounted for in analysis	Statistical tests examined changes from pre- to post- intervention	Outcomes are taken multiple times before and after the intervention	Statistical analysis accounted for individual-level data	Overall rating
**Abrams et al., 2011**	√	√	X	X	X	√	√	X	√	√	X	NA	**Poor**
**Harada et al., 2016**	√	X	?	?	?	√	√	?	?	√	X	NA	**Poor**
**Mehrotra et al., 2014**	√	√	√	√	X	√	√	√	√	√	X	NA	**Fair**

√ - Yes. X – No. ? – Not reported by the authors in the original manuscript. NA – Not applicable.

Table S3. NHLBI Study Quality Assessment Tool for Observational Cohort and Cross-sectional Studies.

	Research objective stated	Clearly specified study population	≥50% participation of eligible persons	Subjects recruited from similar populations	Sample size justification provided	Exposure measured before outcome	Sufficient timeframe	Different levels of exposures examined	Defined, valid, reliable, and consistent exposure	Exposures assessed more than once over time	Defined, valid, reliable, and consistent outcome	Blinded outcome assessors	Loss to follow-up ≤20%	Statistically adjusted confounding variables	Overall Rating
**Ba et al., 2021**	✓	✓	✓	✓	?	✓	✓	X	✓	X	✓	?	?	✓	**Fair**
**Htun et al., 2018**	✓	✓	?	✓	?	X	X	X	✓	X	✓	?	NA	✓	**Poor**
**Lee DH et al., 2019**	✓	✓	✓	✓	?	✓	✓	✓	✓	X	✓	✓	✓	✓	**Good**
**Lee KW et al., 2019**	✓	✓	X	✓	?	✓	✓	X	✓	X	X	?	✓	✓	**Poor**
**Meneses et al., 2020**	✓	✓	?	✓	?	X	X	X	X	NA	✓	?	NA	X	**Poor**
**Nanri et al., 2017**	✓	✓	✓	✓	?	✓	✓	X	✓	X	✓	?	?	✓	**Fair**
**Okada et al., 2019**	✓	✓	X	✓	?	X	X	X	X	X	✓	?	NA	✓	**Poor**
**Osonoi et al., 2016**	✓	✓	✓	✓	?	X	X	X	✓	X	✓	?	NA	✓	**Poor**
**Pounis et al., 2013**	✓	✓	✓	✓	?	X	X	✓	✓	X	✓	?	NA	✓	**Poor**
**Uchiyama et al., 2022**	✓	✓	?	✓	?	X	X	✓	✓	X	✓	?	NA	?	**Poor**
**Weikert et al., 2005 (EPIC)**	✓	✓	✓	✓	?	✓	✓	✓	✓	X	✓	?	✓	✓	**Good**

√ - Yes. X – No. ? – Not reported by the authors in the original manuscript. NA – Not applicable.

Table S4. NHLBI Study Quality Assessment Tool for Case-Control Studies.

	Objective research question	Clearly specified eligibility and selection criteria	Sample size justification	Controls recruited from similar population that gave rise to cases	Valid, reliable, consistent inclusion and exclusion criteria, selection of	Cases were clearly defined and differentiated from controls	Cases and controls randomly selected from eligible subjects	Use of concurrent controls	Confirmation that exposure occurred prior to development of the condi-	Clearly defined, valid, reliable, consistent exposure measures	Blinded assessors	Statistically adjusted key confounding variables	Overall rating
**Weikert et al., 2005 (CORA)**	√	√	?	√	√	√	√	√	?	√	?	√	**Fair**

√ - Yes. X – No. ? – Not reported by the authors in the original manuscript.

Table S5. Change in Systolic Blood Pressures (mmHg).

Author, Year	Study type and design	Length of study intervention	Control group	Change^	Main effect of time p-value	Intervention group	Change	Main effect of time p-value	Group by time effect p-value
Agrawal et al., 2010	RCT, Parallel	3 months	Biscuits with ajwain	6.9	<0.05	Biscuits with *Pleurotus Spp.*	-5.1	<0.05	<0.001
Harada et al., 2016	Exp, Single-arm	2 weeks	Baseline	NA	NA	5 g/d *Grifola garga*l	-0.2	NS	NA
Maruyama et al., 2021	RCT, Parallel	6 months	Partial Japanese Diet	-4	NR	Japanese diet (40 ± 33 g/d)	-5	NR	0.94
Poddar et al., 2013%	RCT, Parallel	6 months WL, 6 months WM	90% lean beef 3 d/week	5.5 ± 27.4	NR	8 oz *Agaricus bisporus* 3 d/week	-5.9 ± 11.2	NR	0.15

^Data are presented as mean change and standard deviation (when applicable). The mean change was estimated if baseline and post values only were reported. % Results reported for the first 6 month period during the weight loss phase. Abbreviations: NA: not applicable; NS: not significant; NR: not reported; WL: weight loss; WM: weight maintenance.

Table S6. Change in Diastolic Blood Pressures (mmHg).

Author, Year	Study type and design	Length of study intervention	Control group	Change^	Main effect of time p-value	Intervention group	Change	Main effect of time p-value	Group by time effect p-value
Agrawal et al., 2010	RCT, Parallel	3 months	Biscuits with ajwain	4	<0.05	Biscuits with *Pleurotus Spp.*	-2.1	<0.05	<0.02
Harada et al., 2016	Exp, Single-arm	2 weeks	Baseline	NA	NA	5 g/d *Grifola garga*l	-1.6	NS	NA
Maruyama et al., 2021	RCT, Parallel	6 months	Partial Japanese Diet	-3	NR	Japanese diet (40 ± 33 g/d)	-3	NR	0.98
Poddar et al., 2013%	RCT, Parallel	6 months WL, 6 months WM	90% lean beef 3 d/week	-1.8 ± 11.8	NR	8 oz *Agaricus bisporus* 3 d/week	-3.7 ± 9	NR	0.61

^Data are presented as mean change and standard deviation (when applicable). The mean change was estimated if baseline and post values only were reported. % Results reported for the first 6 month period during the weight loss phase. Abbreviations: NA: not applicable; NS: not significant; NR: not reported; WL: weight loss; WM: weight maintenance.

Table S7. Change in Total Cholesterol (mg/dL).

Author, Year	Study type and design	Length of study intervention	Control group	Change^	Main effect of time p-value	Intervention group	Change	Main effect of time p-value	Group by time effect p-value
Abrams et al., 2011	Exp, Single-arm	8 weeks	Baseline	NA	NA	15 g/d dried *Pleurotus ostreatus*	-1.7 (-17.4, 14)	NS	NA
Agrawal et al., 2010	RCT, Parallel	3 months	Biscuits with ajwain	61	<0.005	Biscuits with *Pleurotus Spp.*	-29	<0.05	<0.001
Harada et al., 2016	Exp, Single-arm	2 weeks	Baseline	NA	NA	5 g/d *Grifola gargal*	-8	NS	NA
Maruyama et al., 2021	RCT, Parallel	6 months	Partial Japanese Diet	-1	NR	Japanese diet (40 ± 33 g/d)	-11	NR	0.033
Poddar et al., 2013%	RCT, Parallel	0-6 months WL	90% lean beef 3 d/week	2 ± 27.6	NR	8 oz *Agaricus bisporus* 3 d/week	-1.7 ± 16.5	NR	0.485
		6-12 months WM		-0.6 ± 28.3			-4.7 ± 37.6		0.603
Spim et al., 2021	RCT, Parallel	66 days	Placebo	3.9	NR	3.5 g/d dried *L. edodes*	1.4	NR	0.5976
Schneider et al., 2011	RCT, Parallel	21 days	Placebo	-6 ± 28	0.504	Soup with 30 g dried *Pleurotus ostreatus*	-18 ± 27	0.059	0.335

^Data are presented as mean change and variance (SD or 95% confidence interval, when applicable). The mean change was estimated if baseline and post values only were reported. % Results reported for the first 6 month period during the weight loss phase. Abbreviations: NA: not applicable; NS: not significant; NR: not reported; WL: weight loss; WM: weight maintenance.

Table S8. Change in HDL Cholesterol (mg/dL).

Author, Year	Study type and design	Length of study intervention	Control group	Change^	Main effect of time p-value	Intervention group	Change	Main effect of time p-value	Group by time effect p-value
Abrams et al., 2011	Exp, Single-arm	8 weeks	Baseline	NA	NA	15 g/d dried *Pleurotus ostreatus*	2.6 (-0.1, 5.2)	NS	NA
Agrawal et al., 2010	RCT, Parallel	3 months	Biscuits with ajwain	-7.7	<0.005	Biscuits with *Pleurotus Spp.*	3.5	<0.05	<0.001
Harada et al., 2016	Exp, Single-arm	2 weeks	Baseline	NA	NA	5g/d *Grifola garga*l	2.8	NS	NA
Maruyama et al., 2021	RCT, Parallel	6 months	Partial Japanese Diet	1	NR	Japanese diet (40 ± 33 g/d)	-1	NR	0.25
Mehrotra et al., 2014	RCT, Parallel (pre vs. post)	16 weeks	Baseline	NA	NA	100 g/d UV treated mushrooms (500 IU D_2)	-1	NS	0.29
						100 g/d UV treated mushrooms (2600 IU D_2)	1		
						100 g/d untreated mushrooms + 1200 IU D_3 capsules	-2		
						100 g/d untreated mushrooms + 7300 IU D_3 capsules	0		
Poddar et al., 2013	RCT, Parallel	0-6 months WL	90% lean beef 3 d/week	NR	NA	8 oz *Agaricus bisporus* 3 d/week	1.7	NR	0.195
		0-12 months WL+WM					4.7		0.007
Spim et al., 2021	RCT, Parallel	66 days	Placebo	-4.3	NR	3.5 g/d dried *L. edodes*	-2.4	NR	0.4335
Schneider et al., 2011	RCT, Parallel	21 days	Placebo	-2.3 ± 6.2	0.279	Soup with 30g dried *Pleurotus ostreatus*	-1.2 ± 6.6	0.571	0.705

^Data are presented as mean change and variance (SD or 95% confidence interval, when applicable). The mean change was estimated if baseline and post values only were reported. Abbreviations: NA: not applicable; NS: not significant; NR: not reported; UV: ultraviolet; WL: weight loss; WM: weight maintenance.

Table S9. Change in LDL Cholesterol (mg/dL).

Author, Year	Study type and design	Length of study intervention	Control group	Change^	Main effect of time p-value	Intervention group	Change	Main effect of time p-value	Group by time effect p-value
Abrams et al., 2011	Exp, Single-arm	8 weeks	Baseline	NA	NA	15 g/d dried *Pleurotus ostreatus*	6.9 (-9.3, 23.1)	NS	NA
Agrawal et al., 2010	RCT, Parallel	3 months	Biscuits with ajwain	5	<0.02	Biscuits with *Pleurotus Spp.*	-6.1	<0.05	<0.001
Harada et al., 2016	Exp, Single-arm	2 weeks	Baseline	NA	NA	5g/d *Grifola gargal*	-6.4	NS	NA
Maruyama et al., 2021	RCT, Parallel	6 months	Partial Japanese Diet	1	NR	Japanese diet (40 ± 33 g/d)	-8	NR	0.043
Poddar et al., 2013	RCT, Parallel	6 months WL	90% lean beef 3 d/week	1.3 ± 24	NR	8 oz *Agaricus bisporus* 3 d/week	-1.2 ± 18.5	NR	0.611
		6 months WM		-5 ± 30.4			-6.7 ± 33.2		0.82
Spim et al., 2021	RCT, Parallel	66 days	Placebo	-5.2	NR	3.5 g/d dried *L. edodes*	7.4	NR	0.3041
Schneider et al., 2011	RCT, Parallel	21 days	Placebo	-1.9 ± 20.1	0.762	Soup with 30g dried *Pleurotus ostreatus*	-8.9 ± 19.3	0.180	0.450

^Data are presented as mean change and variance (SD or 95% confidence interval, when applicable). The mean change was estimated if baseline and post values only were reported. Abbreviations: NA: not applicable; NS: not significant; NR: not reported; WL: weight loss; WM: weight maintenance.

Table S10. Change in Triglycerides (mg/dL).

Author, Year	Study type and design	Length of study intervention	Control group	Change^	Main effect of time p-value	Intervention group	Change	Main effect of time p-value	Group by time effect p-value
Abrams et al., 2011	Exp, Single-arm	8 weeks	Baseline	NA	NA	15 g/d dried *Pleurotus ostreatus*	-63 (-120.9, -5.1)	<0.05	NA
Agrawal et al., 2010	RCT, Parallel	3 months	Biscuits with ajwain	80.5	<0.02	Biscuits with *Pleurotus Spp.*	-53.3	<0.02	<0.001
Maruyama et al., 2021	RCT, Parallel	6 months	Partial Japanese Diet	-3	NR	Japanese diet (higher intake of mushrooms)	-17	NR	0.023
Mehrotra et al., 2014	RCT, Parallel (pre vs. post)	16 weeks	Baseline	NA	NA	100 g/d UV treated mushrooms (500 IU D_2)	21	<0.05	0.05
						100 g/d UV treated mushrooms (2600 IU D_2)	12	NS	
						100 g/d untreated mushrooms + 1200 IU D_3 capsules	55		
						100 g/d untreated mushrooms + 7300 IU D_3 capsules	-24		
Poddar et al., 2013	RCT, Parallel	0-6 months WL	90% lean beef 3 d/week	NR	NA	8 oz *Agaricus bisporus* 3 d/week	-19.3	0.007	NA
		0-12 months WL+WM					-19.3	0.009	
Spim et al., 2021	RCT, Parallel	66 days	Placebo	63.2	NR	3.5 g/d dried *L. edodes*	-18.2	NR	0.0352
Schneider et al., 2011	RCT, Parallel	21 days	Placebo	31 ± 30.1	0.011	Soup with 30g dried *Pleurotus ostreatus*	-38.1 ± 40.7	0.015	<0.001

^Data are presented as mean change and variance (SD or 95% confidence interval, when applicable). The mean change was estimated if baseline and post values only were reported. Abbreviations: NA: not applicable; NS: not significant; NR: not reported; UV: ultraviolet; WL: weight loss; WM: weight maintenance.

Table S11. Change in Fasting Glucose (mg/dL).

Author, Year	Study type and design	Length of study intervention	Control group	Change^	Main effect of time p-value	Intervention group	Change	Main effect of time p-value	Group by time effect p-value
Abrams et al., 2011	Exp, Single-arm	8 weeks	Baseline	NA	NA	15 g/d dried *Pleurotus ostreatus*	-0.1 (-6.3, 6.0)	NS	NA
Agrawal et al., 2010	RCT, Parallel	3 months	Biscuits with ajwain	84.7	<0.005	Biscuits with *Pleurotus Spp.*	-100.9	<0.005	<0.001
Maruyama et al., 2021	RCT, Parallel	6 months	Partial Japanese Diet	0	NR	Japanese diet (higher intake of mushrooms)	-1	NR	0.98
Poddar et al., 2013	RCT, Parallel	0-6 months WL	90% lean beef 3 d/week	NR	NA	8 oz *Agaricus bisporus* 3 d/week	-4.1	0.040	NA
		0-12 months WL+WM					1.5	0.739	
Spim et al., 2021	RCT, Parallel	66 days	Placebo	-1.3	NR	3.5 g/d dried *L. edodes*	1.6	NR	0.4993

^Data are presented as mean change and variance (SD or 95% confidence interval, when applicable). The mean change was estimated if baseline and post values only were reported. Abbreviations: NA: not applicable; NS: not significant; NR: not reported; WL: weight loss; WM: weight maintenance.

Table S12. Change in HbA1c (%).

Author, Year	Study type and design	Length of study intervention	Control group	Change^	Main effect of time p-value	Intervention group	Change	Main effect of time p-value	Group by time effect p-value
Agrawal et al., 2010	RCT, Parallel	3 months	Biscuits with ajwain	1.4	<0.005	Biscuits with *Pleurotus Spp.*	-1.0	<0.05	<0.005
Maruyama et al., 2021	RCT, Parallel	6 months	Partial Japanese Diet	0.1	NR	Japanese diet (higher intake of mushrooms)	0.1	NR	0.23
Mehrotra et al., 2014	RCT, Parallel (pre vs. post)	16 weeks	Baseline	NA	NA	100 g/d UV treated mushrooms (500 IU D_2)	0.1	NS	0.62
						100 g/d UV treated mushrooms (2600 IU D_2)	0.1		
						100 g/d untreated mushrooms + 1200 IU D_3 capsules	0		
						100 g/d untreated mushrooms + 7300 IU D_3 capsules	-0.1		

^Data are presented as mean change and variance (SD or 95% confidence interval, when applicable). The mean change was estimated if baseline and post values only were reported. Abbreviations: NA: not applicable; NS: not significant; NR: not reported; UV: ultraviolet.

Table S13. Change in hs-CRP (mg/dL).

Author, Year	Study type and design	Length of study intervention	Control group	Change^	Main effect of time p-value	Intervention group	Change	Main effect of time p-value	Group by time effect p-value
Dai et al., 2015#	RCT, Parallel	4 weeks	Baseline	NA	NA	5 and 10 g/d dried *L. edode*s	-0.3	0.008	NA
Maruyama et al., 2021	RCT, Parallel	6 months	Partial Japanese Diet	0	NR	Japanese diet (higher intake of mushrooms)	0	NR	0.92
Poddar et al., 2013	RCT, Parallel	6 months WL	90% lean beef 3 d/week	1.3 ± 4.7	NR	8 oz *Agaricus bisporus* 3 d/week	-1.2 ± 3.8	NR	0.015

^Data are presented as mean change and variance (SD or 95% confidence interval, when applicable). The mean change was estimated if baseline and post values only were reported. #Data from 5 and 10 g/d groups were pooled for analysis. Abbreviations: NA: not applicable; NS: not significant; NR: not reported; WL: weight loss.
